# DrugReX: an explainable drug repurposing system powered by large language models and literature-based knowledge graph

**DOI:** 10.21203/rs.3.rs-6728958/v1

**Published:** 2025-06-16

**Authors:** Liang-Chin Huang, Hunki Paek, Kyeryoung Lee, Ediz Calay, Deepak Pillai, Nneka Ofoegbu, Bin Lin, Hua Xu, Xiaoyan Wang

**Affiliations:** 1 IMO Health, Rosemont, IL, 60018, USA; 2 Department of Biomedical Informatics and Data Science, Yale School of Medicine, New Haven, CT, 06510, USA; 3 Department of Health Policy and Management, Tulane University, New Orleans, LA, 70118, USA

## Abstract

Drug repurposing offers a time-efficient and cost-effective approach for therapeutic development by finding new uses for existing drugs. Additionally, achieving explainability in drug repurposing remains a challenge due to the lack of transparency in decision-making processes, hindering researchers’ understanding and trust in the generated insights. To address these issues, we present DrugReX, a system integrating a literature-based knowledge graph, embedding, scoring system, and explanation modules using large language models (LLMs). We validated DrugReX on 15 established drug repurposing cases, achieving significantly high scores. As a real-world use case, we applied DrugReX to identify candidate drugs for Alzheimer’s disease and related dementias (ADRD) and thoroughly evaluated the pipeline. The system identified 25 promising candidates, with nine clustering with FDA-approved ADRD drugs and 10 linked to ongoing clinical trials. For explainability, an LLM was employed to generate explanations supported by evidence from the literature-based knowledge graph. Domain expert evaluation revealed that DrugReX-produced explanations were superior in quality and clarity than using an LLM alone, enhancing the explainability of repurposing predictions. This study represents the first integration of LLMs to provide explainable insights into drug repurposing, bridging computation precision with explainability, and thus, ultimately enabling more transparent and reliable decision-making in therapeutic development.

## Introduction

The development of new therapeutic agents is a long, financially intensive, and complex process, often taking more than ten years and exceeding a billion dollars in investment to bring a single drug to the market^1^. In this challenging landscape, the strategy of drug repurposing, identifying new therapeutic applications for approved or investigational drugs, presents a promising shortcut^2^. Drug repurposing offers several advantages over traditional drug development, including reduced development times, lower costs, and improved success rates, given the existing safety profiles of the repurposed drug. The significance of the drug repurposing strategy has been particularly highlighted during the COVID-19 pandemic, as numerous repurposed drugs received emergency authorization from the FDA^3,4^.

Drug repurposing has advanced through various techniques that have been employed, including target-based methods^5,6^, gene expression-based methods^7–11^, network-based methods^12–14^, and knowledge graph-based methods^15–19^. Despite their success and promise, siloed data sources, limited drug-indication information, and inefficient and non-validated modeling remain the major challenges^20^. The availability of big data and advanced computational techniques has opened a new era in drug repurposing. Biomedical knowledge graphs, such as Wikidata^21^, PrimeKG^22^, and SPOKE^23^, arbitrate an accelerated course to precision medicine and drug repurposing by interlocking comprehensive biomedical knowledge and ensuring compatibility across data sources. However, the challenge remains in developing a comprehensive, scalable, and, especially, explainable system that can navigate the complexities of biomedical information to identify promising repurposing candidates. Current systems often lack transparency and explainability in their decision-making processes, making it difficult for researchers to understand and trust the generated insights. This opacity is a significant barrier to the adoption of computational drug repurposing methods in clinical research and practice.

In response to these challenges, our study introduces a novel system ”DrugReX” that leverages literature-based knowledge graph technology with advanced large language models (LLMs). Our system is designed to be explainable, leveraging the structured nature of knowledge graphs and the interpretative capabilities of LLMs to provide clear explanation for the rationale behind drug repurposing recommendations. We have chosen Alzheimer’s disease and related dementias (ADRD) as a use case in this study, given the urgent need for new therapeutic options in this area. ADRD conditions burden millions and are expected to rise in prevalence^24^. In addition, the complex pathology of ADRD and the need for rapid therapeutic development heighten the demand for innovative research strategies^25^. In this study, we present an innovative system for drug repurposing. Validated against successful drug repurposing and applied to the pressing challenge of ADRD, our system contributes to the potential for identifying new therapeutic opportunities and elucidating the underlying mechanisms of actions. As the biomedical community continues to grapple with the complexities of drug discovery and repurposing, our work offers a promising pathway forward, leveraging the power of advanced computational techniques to unlock new possibilities in the quest for effective treatments.

## Results

### Framework of explainable drug repurposing system

**Figure 1.**
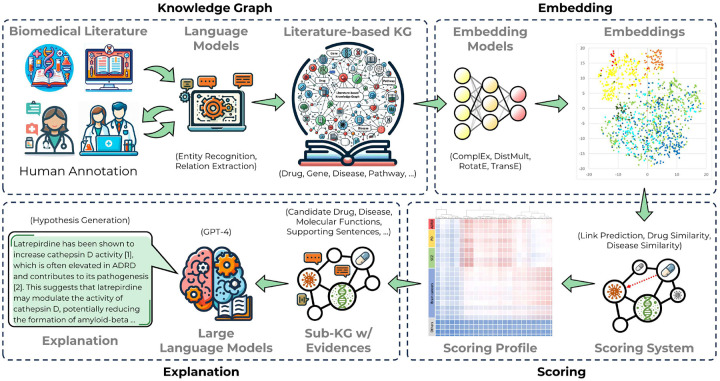
Four modules of our proposed explainable drug repurposing system ”DrugReX”.

This study aims to construct an explainable drug repurposing system ”DrugReX” by leveraging a literature-based knowledge graph, as illustrated in Fig. 1. This system consists of four modules: (i) knowledge graph module, (ii) embedding module, (iii) scoring module, and (iv) explanation module. We first assembled a comprehensive corpus by extracting biomedical literature, including both abstracts and full texts. Next, we utilized the rule-based natural language processing (NLP) tool to construct a biomedical knowledge graph from the obtained entities and relationships. To distill complex, high-dimensional data, we applied knowledge graph embedding models and assessed their performance using three pertinent metrics for potential machine learning applications. Building on this, we crafted a scoring system for drug repurposing that incorporated three integral scores: Link Prediction Score, Drug Similarity Score, and Disease Similarity Score. Alzheimer’s disease and related dementias (ADRD) were selected as a case study which entailed calculating the Repurposing Score (R-score) between each drug and the designated disease. Lastly, we employed GPT-4, a state-of-the-art language model, to delineate hypotheses that elucidate the potential of candidate drugs for treating ADRD, utilizing a sub-knowledge graph that connects each candidate drug with ADRD and their mutual molecular function neighbors.

### Literature-based knowledge graph

We identified more than 100 million relationships across five entity types and ten biomedical relation types employed in our study. Generic concepts such as ”Disease” (CUI: C0012634) were subsequently eliminated from these relationships. Subsequently, we refined the relationships utilizing the optimal model amongst the six BERT-based NLP models we trained, specifically PubMedBERT. This model exhibited an F_1_ score of 0.808, with detailed performances illustrated in Supplementary Table S1. Consequently, we constructed a literature-based knowledge graph (LitKG), comprising 282 thousand entities, all of which are detailed in Supplementary Table S2. A total of 25 million relations, presented in Supplementary Table S3, were extracted from 66 million sentences derived from the literature.

### Using LitKG for drug repurposing

**Figure 2.**
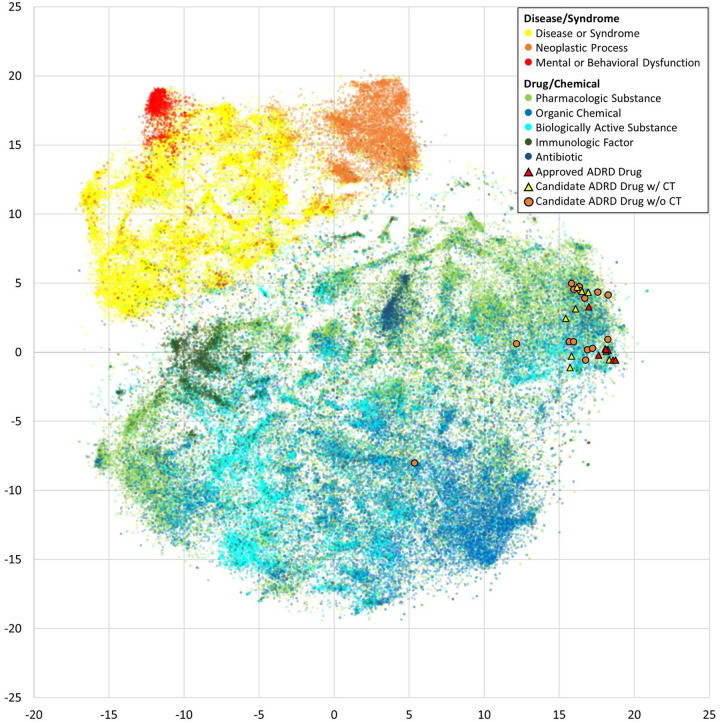
Visualization of drug and disease entities embedded in a 2D space. ADRD: Alzheimer’s disease and related dementias; w/ (w/o) CT: with (without) clinical trials for treating ADRD.

We developed the scoring system for drug repurposing on considering drug-disease link prediction (LPS), drug similarity (DrSS), and disease similarity (DiSS). Each of these three scores were computed using trained knowledge graph embedding models. A range of different embedding algorithms were deployed to train these models, with their respective performances evaluated based on a 10-fold cross-validation. Supplementary Table S4 indicates that TransE, regularized by L1 normalization, outperforms in four out of five measurements (MR = 1.697, MRR = 0.833, Hits@1 = 0.730, and Hits@10 = 0.990). Figure 2 further demonstrates that the embeddings effectively distinguish between drug and disease entities within a two-dimensional space. Based on these results, the TransE model was selected for further analyses. Finally, we calculated the R-scores for all 1.7 billion drug-TREATS-disease triples, encompassing 48,461 Pharmacologic Substance and 35,616 Disease/Syndrome interactions. The distribution of the scores for LPS, DrSS, DiSS, and R-score are visually depicted in Supplementary Fig. S1a.

### Evaluation of the scoring system

Firstly, we assessed the efficacy of the scoring system through an examination of the scores of drug-TREATS-disease triples that exist in the LitKG in contrast to those that do not. Supplementary Fig. S1b-e suggests that, across all scoring types (LPS, DrSS, DiSS, and R-score), the drug-TREATS-disease relations found in the LitKG have higher scores compared to those absent in the LitKG (average R-score = 0.626 vs. 0.352; Supplementary Fig. S1e). This observation aligns with our expectations when we consider the relationships existing in LitKG as the benchmark. Secondly, we examined 15 successful drug repurposing instances^26^ as illustrative examples, assessing each for its R-score. To establish an unbiased perspective, we disregarded any relations between successful drug-disease instances and subsequent treatment relations during the training period of the knowledge graph embedding models. As displayed in Table 1, the R-score of these 15 successful repurposing instances ranged between 0.575 to 0.711. Notably, all R-scores were significantly higher than the average R-score (0.352) with p-values ranged from 0.002 to 0.02 (the p-value was determined based on the R-score distribution spanning all drug-disease pairs). To recreate a scenario wherein the experiments were conducted prior to the drugs’ new indications getting approval or even before the initiation of phase 3 clinical trials, we utilized a time series split method to verify the discoveries. Among the 15 successful drug repurposing instances, 14 unveiled significant R-scores when only factored by the knowledge graphs constructed before their approval dates, with the adjusted p-value (Adj PV^*a*^) ranging between 1.9E-5 and 0.0215, as referenced in Table 1. Our exploration of Thalidomide’s initial insignificant adjusted p-value (0.4971) traced this back to low drug and disease similarity scores during the early stages. However, moving the time-split point to the phase 3 trials stage revealed a significant score for treating erythema nodosum leprosum, highlighting the influence of high link prediction scores on the R-score when drug and disease similarity scores are absent. Furthermore, out of 11 successful drug repurposing instances that were documented in the literature pre-phase 3 clinical trials (excluding four drugs that were absent), 10 still conveyed significant R-scores, with adjusted p-value (Adj PV^3^ or Adj PV^*a*−4^) from 1.6E-5 to 0.0353. This evaluation outcome implies that the scoring system can generate plausible scores for successful drug repurposing instances. Specific insights yielded from the drug repurposing instances indicate the potential utility of this scoring system in prospective pharmaceutical developments and validations.

### Drug repurposing for Alzheimer’s disease and related dementia

**Figure 3.**
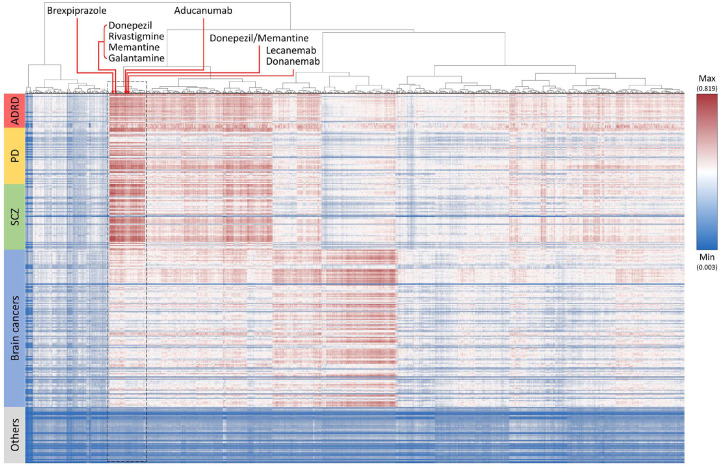
Clustering analysis of drug repurposing outcomes. FDA-approved ADRD drugs are indicated by red arrows. PD: Parkinson’s disease; SCZ: schizophrenia.

We computed all R-scores between 48,461 drugs and 327 diseases/proteins/symptoms. As an efficiency measure aimed at mitigating the computational demand inherent in performing a clustering analysis, we retained only one drug with the highest R-score for ADRD from each drug category, based on the UMLS hierarchy. Consequently, a pool of 8,716 drugs remained for subsequent clustering analysis. Our analysis grouped these 8,716 drugs into several clusters exhibiting similar R-score patterns. The drug cluster, as depicted in Fig. 3, predominantly exhibits an R-score profile with high score for diseases such as ADRD, Parkinson’s disease, and schizophrenia, while demonstrating lower scores for brain cancers and other diseases. Noteworthy, all extant FDA-approved drugs for Alzheimer’s disease treatment (i.e. aducanumab, lecanemab, and donanemab^27,28^) or symptom management (i.e. brexpiprazole, donepezil, rivastigmine, memantine, galantamine, and donepezil/memantine^28^) were grouped in this cluster, with drugs for treating the disease and those for managing its symptoms further divided into two clades. Similarly, we observed that approved drugs for brain cancers^29^ formed a distinct cluster (Supplementary Fig. S2). Based on these findings, we subsequently selected 25 candidate drugs that were in the same clade as these FDA-approved Alzheimer’s disease drugs for further analysis (Table 2). In the scoring profile, the 25 drug candidates grouped not only with the nine FDA-certified drugs but also in a 2D space as per the embedding outcome (Fig. 2; exceptions: lemon oil and ITI-214). Importantly, all the candidates had a significantly elevated R-score (Table 2; p-values ranged from 8.1E-6 to 0.0082; adjusted p-value for those have clinical trial for treating ADRD ranged from 0.0005 to 0.0059). Furthermore, each candidate was closely related to at least one FDA-approved drug (Table 2; Pearson correlation coefficients to the closest ADRD drug were from 0.9871 to 0.9976 based on the scoring profile). Among these 25 candidate drugs, ten of them have completed/terminated clinical trials for treating ADRD (Supplementary Fig. S3 and Data S1; source: ClinicalTrials.gov and a review paper^30^). They are latrepirdine, lithium, mecamylamine, phenserine, pimavanserin, acamprosate, baclofen, psilocybin, rasagiline mesylate, and ST-101. The first five of them demonstrate at least one relation with AD-related proteins/symptoms according to the LitKG, whereas the remaining five exhibit no such connection. The potential of these ten selected candidates, elucidated through the use of large language models, is described in the following section.

### Explaining drug repurposing outcomes by GPT-4

**Figure 4.**
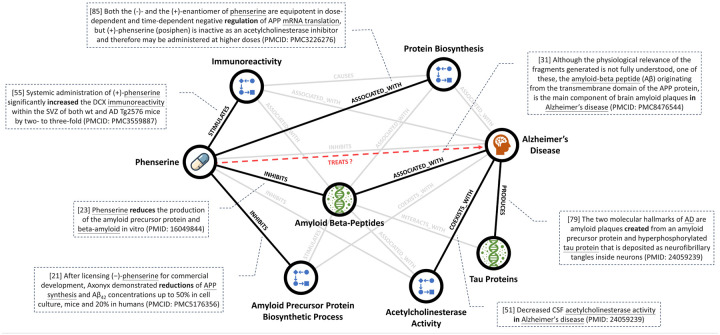
Sub-knowledge graph illustrating supporting sentences for a sample candidate drug for ADRD. In this sub-knowledge graph, nodes (circles) represent entities, edges denote relationships, and dotted rectangles enclose supporting sentences. Black edges indicate relationships for which supporting sentences were input and cited by GPT-4 in generating a hypothesis, whereas gray edges represent relationships where supporting sentences were input but not cited. Within the supporting sentence rectangles, entities (subjects and objects) are underlined, and relationships are emphasized in bold. The reference index for supporting sentences cited by GPT-4 is shown in brackets, followed by the corresponding source identifiers (PMCID or PMID) in parentheses.

Supporting sentences gathered from the sub-knowledge graph, constructed by each candidate drug, ADRDs, AD-related proteins/symptoms, and mutual molecular function neighbors are available in Supplementary Data S2. Figure 4 illustrates the sub-knowledge graph for an example candidate drug, phenserine, along with supporting sentences that define the relationships between the entities. Table 3 presents representative explanations for phenserine, including hypotheses generated based on various tasks described in Supplementary Table S5. These tasks and generated hypotheses will henceforth be referred to as ”LitKG-GPT4”, ”LitKG’-GPT4”, ”KG-GPT4”, ”GPT4”, and ”Consensus” (a compared GPTs application with search engine for research^31^). Supplementary Data S2 likewise provides the results for the remaining nine candidate drugs. On average, LitKG-GPT4 and LitKG’-GPT4 cited 21.4 and 16.8 sentences respectively, while KG-GPT4 cited 22.6 relations and Consensus cited 2.4 articles. In LitKG’-GPT4 and KG-GPT4, an overwhelming majority of sentences (99.3%) and all relations (100%) were drawn from supporting sentences, rather than unrelated sentences. A review by our domain experts revealed that correctly cited sentences or relations, which align with the meaning and context, constituted 93.0%, 93.5%, and 96.9% correctness of LitKG-GPT4, LitKG’-GPT4, and KG-GPT4, respectively.

**Figure 5.**
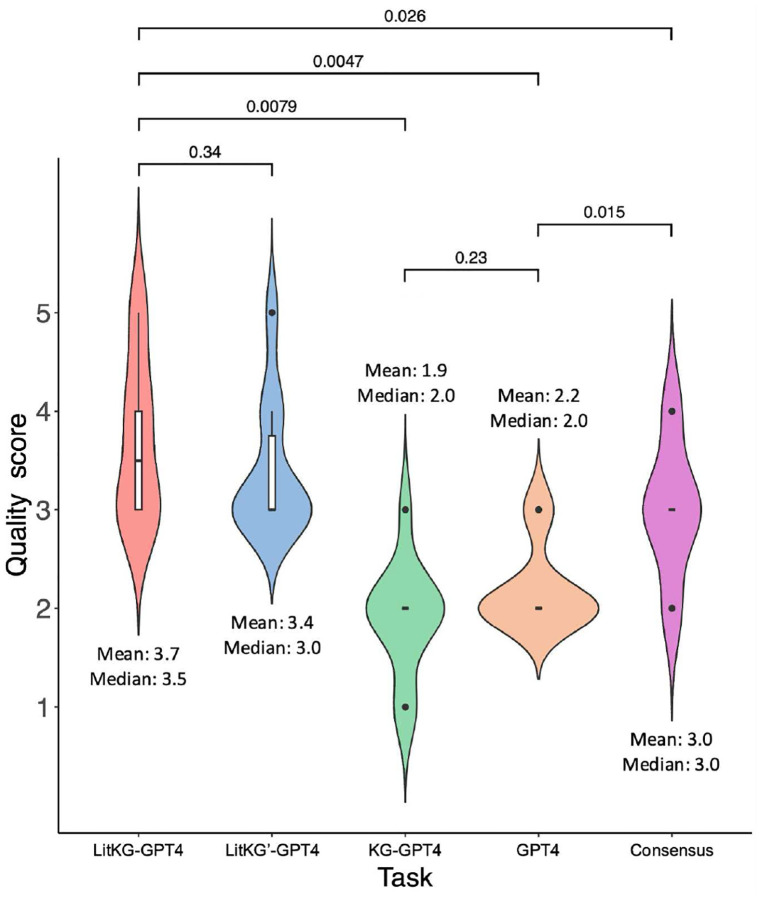
Performances of different hypothesis generation tasks. The p-value of Wilcoxon signed-rank test between each task pair is shown on top.

Besides quantifying performance through correct citation rates, the quality of outcomes from diverse tasks were also assessed by our domain experts. This examination aimed to discern which hypothesis yielded more informative, detailed, and logically inferable mechanisms of action. Each hypothesis received a quality score in the range of 1 (being the worst) to 5 (the best). Subsequent to a comprehensive review, our domain experts revealed that nine out of ten hypotheses generated from the LitKG-GPT4 tasks provided superior explanations for the potential mechanism of action of each candidate drug in treating ADRDs; the rest one was generated from a LitKG’-GPT4 task (quality scores and annotations can also be found in Supplementary Data S2). On average, the quality scores of LitKG-GPT4, LitKG’-GPT4, KG-GPT4, GPT4, and Consensus were 3.7, 3.4, 1.9, 2.2, and 3.0, respectively. Figure 5 demonstrates that the hypotheses generated from LitKG-GPT4 tasks were significantly superior to those from KG-GPT4 tasks (p-value = 0.0079, Wilcoxon signed-rank test), GPT4 tasks (p-value = 0.0047), and Consensus tasks (p-value = 0.026). Additionally, Consensus, a GPTs application with AI-powered academic search engine, demonstrated significantly better performance than GPT-4 along (p-value = 0.015). Conversely, differences between LitKG-GPT4 and LitKG’-GPT4, and between KG-GPT4 and GPT4 outcomes, were not statistically significant (p-values = 0.34 and 0.23, respectively).

## Discussion

In this study, we introduced our innovative drug repurposing system that is comprehensive, scalable, and explainable leveraging the knowledge graphs and the interpretative capabilities of LLMs. Our system consists of four interrelated modules, each addressing a critical aspect of the drug repurposing workflow. First, the literature-based knowledge graph construction module utilized the wealth of information contained within the full-texts PubMed Central articles to enable a comprehensive knowledge graph. This graph is enriched with semantic relationships extracted via advanced NLP tools, such as SemRep and PubMedBERT, enabling a nuanced understanding of drug-disease interactions. Second, the embedding module reduced the dimensionality of data while preserving the essential relationships and patterns, making it possible to navigate and analyze the knowledge graph effectively. Third, the repurposing score module calculates a ”Repurposing Score” for each drug and ranks them based on their potential effectiveness for new indications. This scoring system is based on a combination of graph-based metrics and predictive modeling, providing a quantifiable measure of repurposing viability. Fourth, the LLM-based module elucidates drug action pathways. Our system includes an LLM-based module that generates hypotheses about potential drug action mechanisms. This module interprets the complex interplay of factors involved in drug efficacy and provides detailed, understandable explanations of how a repurposed drug might act against a new disease target.

Drawing upon the assessed judgment of our domain experts, a qualitative analysis was carried out on the hypotheses generated from all aforementioned tasks. Through this analysis, it was observed that the hypotheses generated from LitKG-GPT4 or LitKG’-GPT4 tasks surpassed those from KG-GPT4, GPT4, or Consensus tasks in quality. This finding suggests that the GPT-4 model can formulate hypotheses that are marked with a higher degree of logical inference using the supporting sentences from literature-based knowledge graphs. In the GPT4 tasks, the absence of provided evidence within these explanations can pose a challenge for researchers. Tracing back the source of these explanations can prove problematic, and the generated outcomes may potentially arise from GPT-4’s conjectural reasoning or ”hallucination”^32^. In such instances, the lack of concrete evidence within the hypothesis may not necessarily invalidate its accuracy, but it does limit its verifiability. Thus, while the GPT4 task-generated hypotheses provide thoughtful and intricate theories, their value is partly compromised by the difficulties in tracing back their derivation, highlighting the model’s tendency towards ”hallucinatory” outcomes.

While artificial intelligence and machine learning, particularly deep learning, are revolutionizing various sectors with their high accuracy, their ”black box” nature raises issues about explaining decision-making mechanisms^33,34^, particularly in sensitive fields, prompting a growing interest and research in explainable AI (XAI) to address transparency and explainability questions^35^. Attention layers in deep learning models, typically found in transformer neural networks, are integral for sequence modeling as they adjust the importance of input values via a weighted mean reduction process to increase feature weight explanation^36^. Previous studies implement explainable drug repurposing models via various methods, including mathematical procedures^37^, knowledge graph embeddings^38^, graph neural networks^39^, deep learning-based path-reasoning frameworks^40^, and multi-hop reasoning approaches^41^, providing insight into drug-disease associations and the mechanism of drug action. While these works offer varying degrees of explainability for input features, our model differentiates itself by focusing on elucidating the predicted outcome and the potential biological mechanism of action of candidate drugs, offering a comprehensive understanding of drug repurposing and effectiveness.

A previous study presented a ”semantic multi-layer guilt-by-association” approach for repurposing drugs for Alzheimer’s disease utilizing biomedical knowledge graphs, aiming to overcome challenges like gene dominance and a small number of drug-disease entities by leveraging similar gene function principles^42^. This study proposed ten candidate drugs for treating Alzheimer’s disease and applied their model to seven drugs in phase III clinical trials for Alzheimer’s disease^43^. These 17 drugs shown in our knowledge graph embedding space and their R-scores for ADRD are available in Supplementary Fig. S4 and Table S6, respectively. Upon comparison, we discovered that the ten candidate drugs suggested by the previous study cluster together with considerably high R-scores (with p-values ranging from 0.0002 to 0.0040 and adjusted p-values ranging from 0.0002 to 0.0033), though they may share similar mechanisms. According to our scoring profile, brexpiprazole is the closest ADRD drug for nine of them. Despite some drugs (caffeine, hydralazine, metformin, and omega-3 carboxylic acids) not being closely related to FDA-approved ADRD drugs in the embedding space, they show potential for treatment and are undergoing phase III clinical trials for ADRD. Additionally, two candidates proposed in our study, lemon oil and ITI-214, are not closely related to FDA-approved ADRD drugs. Lemon oil, employed in aromatherapy, has shown potential in treating Alzheimer’s disease by improving cognitive function and personal orientation, including a reduction in acetylcholinesterase levels and elevation of brain-derived neurotrophic factor, thereby enhancing synaptic plasticity and demonstrating significant benefits^44,45^. ITI-214, a potent and highly specific inhibitor of the PDE1 family, has demonstrated, through its distinct anti-inflammatory and gene regulation effects on microglial cells, potential for treating Alzheimer’s disease and other neurodegenerative diseases associated with inflammation and microglia proliferation, making it a valuable candidate^46,47^.

Through the execution of the DrugReX pipeline, we identified 25 potential ADRD drugs, including ten of which are under clinical trials for ADRD treatment, and we provided potential explanations of their mechanisms of action. Yet, this approach is not without limitations. First, due to computational and memory constraints, we could not cluster all 48,461 drugs across 35,616 diseases when creating the scoring profile. Instead, we built the clustered scoring profile using 8,716 representative drugs and 327 selected diseases/proteins/symptoms. Second, the selection of these 327 entities necessitates expertise in biological knowledge and expert review. Varying choices may yield different clustering results, making this process less straightforward to apply and reproduce automatically for other diseases. Third, in our identification of candidate drugs, we used known ADRD drugs as seeds and their neighbors as candidates. If no FDA-approved drug exists for certain conditions, such as rare diseases, recent research^48^ has focused on addressing this gap by applying network medicine principles that target the molecular mechanisms of disease treatment, a strategy we also considered in our study. In such cases, we are forced to rely solely on the R-score and search for the candidate drug with the highest corresponding R-score. Lastly, while identifying candidate drugs using known and FDA-approved drugs minimizes risk (due to potential similarities in biological pathways or mechanisms of action), this approach may overlook potential candidates not closely related to the current approved drugs. Based on this study’s preliminary findings, future enhancements could position this approach as a powerful asset in the quest for effective treatments for a wide range of diseases.

## Methods

### Corpus preparation

We initially collected 30 million biomedical literature (spanning from 1965 to 31 August 2022) from PubMed, including both titles and abstracts. To broaden the scope of biomedical data, we also obtained 5.6 million full-text articles (published from 1781 to 22 September 2022) from PubMed Central (PMC). To avoid redundancy, our collection from the full-text articles was restricted to the main body, excluding titles, abstracts, and references.

### Knowledge graph construction

We primarily utilized biomedical literature sourced from SemMedDB^49^ (Version 43; accessed on 7/22/2022). SemMedDB employs SemRep^50^ (Version 1.8), a rule-based NLP tool tailored for biomedical texts. Our methodology was designed to augment the extraction of biomedical entities and relations from PubMed abstracts. SemRep was also utilized to extract additional biomedical entities and relations from PMC’s full-text articles. SemRep’s default lexicon is anchored on the 2018 version of the Unified Medical Language System (UMLS), but we upgraded to the 2022 version to encompass emergent biomedical terminologies, including terms such as COVID-19 (UMLS Concept Unique Identifier: C5203670). Finally, we consolidated all results after screening all abstracts and full-text articles using SemRep with the updated lexicon.

To enhance the accuracy of SemRep results, NLP models rooted in BERT were utilized to refine the outcomes. Firstly, two domain experts were enlisted to evaluate the accuracy of results generated by SemRep. Any inconsistencies arising from their evaluation were resolved by a third expert. To obviate any interpretive ambiguities created by SemRep, all relations mentioned in Supplementary Table S7 were normalized prior to evaluation. A minimum of 100 instances were annotated per each type of relation. This, in conjunction with annotations garnered from a prior study^17^, resulted in an additional 8,241 annotations (Supplementary Data S3). Secondly, we implemented a 10-fold cross-validation method to train several NLP models including BERT^51^ (cased and uncased), BioBERT^52^, BioClinicalBERT^53^, BlueBERT^54^, and PubMedBert^55^ in the context of the augmented annotations. The model with the highest performance was subsequently used to sift through SemRep results, filtering out any false relations.

To focus on entities integral to biomedical research and drug discovery, we implemented a two-step noise removal process. First, we dismissed generic concepts as defined by SemMedDB and our team of annotators (Supplementary Data S4). Subsequently, we retained only entities falling within eight semantic groups^56^: Anatomy, Chemicals & Drugs, Devices, Disorders, Genes & Molecular Sequences, Phenomena, Physiology, and Procedures defined by UMLS. These entities were subsequently re-categorized into five distinct entity types, namely, ”Drug/Chemical”, ”Gene/Protein”, ”Disease/Syndrome”, ”Pathway/Function”, and ”Others”. To ensure concise relation types and minimize conflicting relationships between entities, the negated counterparts of relation types (e.g., NEG_INHIBITS) identified by SemRep were excluded from the knowledge graph. Consequently, all downstream analyses and drug-disease relationship predictions involved only positive relations. Finally, our biomedical knowledge graph was constructed employing ten fundamental relation types: ”ASSOCIATED_WITH”, ”CAUSES”, ”COEXIST_WITH”, ”COMPLICATES”, ”INHIBITS”, ”INTERACTS_WITH”, ”MANIFESTATION_OF”, ”PRODUCES”, ”STIMULATES”, and ”TREATS”.

### Knowledge graph embedding models

Knowledge graph embedding simplifies complex, high-dimensional data into lower-dimensional vector space, enabling machine learning tasks and prediction of data relationships, with notable effectiveness in fields such as recommendation systems, drug discovery, and NLP. We divided the knowledge graph randomly into training, validation, and test datasets, maintaining a ratio of 8:1:1. Subsequently, we utilized ComplEx, DistMult, RotatE, and TransE (regularized by either none, L1, or L2 normalization) implemented by DGL-KE^57^ package to train knowledge graph embedding models. We maintained uniformity in the hyperparameters used during the training: batch size (2,070), negative sampling size (30), hidden dimension (400), learning rate (0.1), and maximum step (10,000). The performance of these models was evaluated using three key metrics: mean rank (MR), mean reciprocal rank (MRR), and Hits@k measure. MR signifies the mean rank assigned to the true relation amidst all relations within a test dataset, while MRR is the median inverse rank for the entirety of the test triples. The Hits@k measure represents the percentage of relations where the true triple appears among the top k ranked triples. Ideally, a lower MR and higher MRR and Hits@k denote superior performance. The most effective model as per these metrics will be selected for the future downstream scoring system.

### Scoring system for drug repurposing

We utilized drug repurposing as a representative example to demonstrate the application of literature-based biomedical knowledge graph with LLMs. Our designed Repurposing Score (R-score) essential in prioritizing drugs for a particular disease consists of three key scores: Link Prediction Score (LPS), Drug Similarity Score (DrSS), and Disease Similarity Score (DiSS). The aim of link prediction in a knowledge graph is to estimate the likelihood of currently unidentified relations between entities. The highest performing knowledge graph embedding model, along with its scoring function, was employed to compute the scores for all drug-TREATS-disease triples pertaining to target diseases. In order to make these scores compatible with DrSS and DiSS, we applied a Box-Cox transformation and then normalized these scores into LPS’_*θλ*_ ranging between 0 and 1 for drug *θ* treating disease *λ*. The computation of DrSS was done drawing upon prior knowledge regarding the candidate drugs and target diseases:

(1) $$DrSS_{\theta \lambda }=\frac{\sum_{i=1}^{I}T_{i\lambda }S_{i\theta }}{\sum_{i=1}^{I}T_{i\lambda }},i\neq \theta$$

DrSS_*θλ*_ refers to the basic drug similarity score for drug *θ* treating disease *λ*. T_*iλ*_ equals 1 when the drug-disease treatment relation ”drug i-TREATS-disease *λ*” exists within the knowledge graph and equals 0 when no such relation exists. In scenarios where no drug is available to treat the disease *λ* or where the drug *θ* is the singular treatment option (resulting in a zero denominator), DrSS_*θλ*_ will not be calculated. S_*iθ*_ represents the normalized entity cosine similarity between the drugs *i* and *θ* as per the knowledge embedding model. These cosine similarities are deduced using the scikit-learn package^58^. With regards to all triples of ”drug-TREATS-disease” relevant to target diseases, a Box-Cox transformation is executed, following which these scores are normalized to result in DrSS’_*θλ*_ that range from 0–1. To compute DiSS_*θλ*_, which signifies the raw disease similarity score for drug *θ* treating disease *λ*, following is the scoring function:

(2) $$DiSS_{\theta \lambda }=\frac{\sum_{j=1}^{J}T_{\theta_{j}}S_{\lambda_{j}}}{\sum_{j=1}^{J}T_{\theta_{j}}},j\neq \lambda$$

S_*λ j*_ represents the normalized entity cosine similarity between diseases *λ* and *j*. In cases where no disease is treated by drug *θ* or if the disease *λ* is the singular disease treated by that drug, calculating DiSS_*θλ*_ will be non-applicable. The DiSS scores are then subjected to a Box-Cox transformation followed by normalization, which adjusts these scores into a DiSS’_*θλ*_ value in a range of 0 to 1. The ultimate R-score_*θλ*_ is utilized to predict drug *θ*’s ability to treat disease *λ*. This score is defined as the mean of LPS’_*θλ*_, DrSS’_*θλ*_, and DiSS’_*θλ*_. Should DrSS’_*θλ*_ or DiSS’_*θλ*_ be not calculated because of inadequate initial knowledge about the disease *λ* or drug *θ*, respectively, these values will be omitted while calculating R-score_*θλ*_.

### P-value and adjusted p-value of an R-score

The R-score holds a value within the range of 0 to 1. To determine the significance of an R-score, we initially calculated the R-scores for all pairings between drugs (denoted by the semantic type: ”Pharmacologic Substance”) and diseases (labeled by the entity type: ”Disease/Syndrome”) within the knowledge graph. This was followed by quantifying the count of extreme values that outstripped the compared R-score. Finally, by dividing this count by the total number of drug-disease pairs, we derived the p-value.

The p-value is adjusted to simulate the hypothesis that if drug *θ* is approved for treating disease *λ*, we calculate an R-score and its p-value for the *θλ* pair at a specific time point pre-approval, by implementing a time series split methodology. Initially, we constructed a subset knowledge graph, retaining only the LitKG’s relations reported in literature prior to that specific date. If drug *θ*, disease *λ*, or both are not present in the subset knowledge graph, the adjusted p-value will be denoted as ”NA”. This was followed by reconstructing knowledge graph embeddings, recalculating all R-scores between drugs and diseases, and subsequently calculating the p-value. The adjusted p-value, based on *θλ*’s approval date, is denoted as ”Adj PV^*a*^”. If the p-value is adjusted according to the start date of *θλ*’s phase *n* clinical trial sourced from ClinicalTrials.gov, we term this as ”Adj PV^*n*^”. Should the p-value be adjusted according to the start date of any *θλ*’s clinical trial, we refer to this as ”Adj PV^0^”. There may be instances where *θλ*’s clinical trial data is absent (due to ClinicalTrials.gov being established in 2000); in such cases, the p-value would be adjusted based on a date four years prior to its approval (given that the average duration of phase 3 clinical trials is four years^59^); we refer to this as ”Adj PV^*a*−4^”. By adjusting for the specific circumstances and timeframes related to each drug-disease pair, a more nuanced and accurate evaluation of their relative significance can be achieved.

### Use case: Alzheimer’s disease and related dementias

In this study, Alzheimer’s disease and related dementias (ADRD) served as a case study for drug repurposing via LitKG. Given the absence of the terminology ”Alzheimer’s disease and related dementias” in the present iteration of UMLS, we designated ADRD by applying the ”Alzheimer’s Disease” term (UMLS CUI: C0002395) along with its corresponding child terms as defined by UMLS hierarchy. Additionally, we incorporated proteins related to Alzheimer’s disease (namely tau proteins and amyloid beta-peptides), as well as symptoms such as vasogenic edema and microhemorrhage (referred to as ”AD-related proteins/symptoms” hereafter). Consequently, our LitKG includes a total of 30 ADRD-related terms (”ADRDs” hereafter). For the sake of comparison, we also encompassed brain-related diseases such as Parkinson’s disease (with its 50 subtypes), schizophrenia (58 subtypes), and brain cancers (139 subtypes), as well as 50 diseases unrelated to the brain (the outgroup). By applying knowledge graph embedding, we identified those 50 diseases that have the least cosine similarity to Alzheimer’s disease. A comprehensive list of these 327 diseases/proteins/symptoms is provided in Supplementary Data S5. In determining the R-score between each drug and the disease or symptom, we adopted the aforementioned scoring system. Meanwhile, in calculating the score between each drug and the proteins associated with Alzheimer’s disease, we assessed the score for ”drug-INHIBITS-protein” relations and normalized the outcomes utilizing identical processes. After conducting numerical assessments for all target drug-disease/protein/symptom pairs, we employed Ward’s method^60^ for grouping drugs that exhibit analogous scoring profiles. Potentially efficacious drugs for treating ADRDs are expected to have high scores for treating ADRDs and low scores for treating non-related diseases.

### Designing prompts for explaining drug repurposing outcomes

To explain the potential of candidate drugs for treating ADRD, we employed the LLM, GPT-4, to generate hypotheses. To begin, a sub-knowledge graph was constructed, linking each candidate drug with ADRDs. This graph was designed based on the relations between four kinds of entities: the candidate drug, AD-related proteins/symptoms, ADRDs, and the common molecular function neighbors of both the candidate drug and ADRDs. The designated candidate drug-TREATS-ADRDs relations were purposely omitted. Secondly, we tapped into the preliminary source sentences supporting these relations and used these to formulate prompts for each candidate drug. Templates for these prompts are presented in Supplementary Table S5. A limit was placed on the total number of supporting sentences (task ”LitKG-GPT4”), capped at 50. In instances where there were more than 50 supporting sentences, a random selection of 50 sentences was made. For evaluation, we randomly selected an equal number of unrelated drug-disease sentences (i.e., non-candidate drugs and non-ADRDs) from the LitKG and included them in the prompts (task ”LitKG’-GPT4”). The sequence of supporting sentences and unrelated sentences within the prompts was shuffled. In contrast to the results of using supporting sentences, we also utilized a prompt based solely upon the relation types present in typical non-literature-based knowledge graphs (task ”KG-GPT4”) and another prompt including only the candidate drug’s name and Alzheimer’s disease (tasks ”GPT4” and ”Consensus”, a GPT-4-based application with AI-powered academic search engine). When operating GPT-4, we set the parameters to the following: system = ”You are a neurologist and a professional scientific journal paper writer”, model = ”gpt-4”, and temperature = 0. This approach allowed for a comprehensive, systematic review of potential ADRD therapeutics in a controlled, reproducible manner.

## Supplementary Files

This is a list of supplementary les associated with this preprint. Click to download.

- DrugReXScienti cReportsSupplementary.pdf
- DataS1.xlsx
- DataS2.xlsx
- DataS3.csv
- DataS4.csv
- DataS5.tsv

## Figures and Tables

**Table 1.** R-scores for successful drug repurposing instances.

Drug name	New indication	R-score	P-value	Approval date**	Adj PV^*a*^	Phase 3 date**	Adj PV^3^
Minoxidil	Hair loss	0.6174	0.0066	87/03/03	0.0142	NA	0.0353*
Zidovudine	HIV/AIDS	0.6899	0.0005	87/03/19	0.0094	NA	0.1505*
Sildenafil	Erectile dysfunction	0.6480	0.0026	98/03/27	0.0135	NA	NA
Thalidomide	ENL	0.6348	0.0039	98/07/16	0.4971	NA	0.0035*
Celecoxib	FAP	0.6017	0.0102	99/12/23	0.0215	NA	NA
Atomoxetine	ADHD	0.7112	0.0002	02/11/26	0.0002	00/08/01	0.0015
Duloxetine	SUI	0.6485	0.0025	04/08/03	0.0011	01/02/01	0.0039
Rituximab	Rheumatoid arthritis	0.6223	0.0057	06/02/28	1.9E-5	05/06/30	1.6E-5
Thalidomide	Multiple myeloma	0.6224	0.0057	06/05/25	0.0015	01/12/01	0.0016
Raloxifene	Breast cancer	0.6413	0.0032	07/09/13	0.0005	98/06/01	0.0005
Fingolimod	Multiple sclerosis	0.6775	0.0009	10/09/21	0.0053	06/01/01	NA
Dapoxetine	Premature ejaculation	0.6872	0.0006	12/05/01	0.0037	03/06/01	NA
Topiramate	Obesity	0.5936	0.0126	12/07/17	0.0012	00/07/01	0.0029
Ketoconazole	Cushing syndrome	0.6753	0.0010	14/02/25	0.0023	13/04/24	0.0032
Aspirin	Colorectal cancer	0.5750	0.0200	15/09/14	0.0085	92/02/01	0.0122

*: given the unavailability of the date for the phase 3 clinical trial, the calculated value is derived from Adj PV^*a*−4^.

**: date format: YY/MM/DD. ENL: erythema nodosum leprosum; FAP: familial adenomatous polyps; ADHD: attention-deficit/ hyperactivity disorder; SUI: stress urinary incontinence.

**Table 2.** Candidate drugs’ R-scores for ADRD. The drugs with clinical trials for treating ADRD are highlighted in bold.

Drug name	Original designed indication*	Closest ADRD drug	Correlation	R-score	P-value	First CT for ADRD**	Adj PV^0^
**Phenserine**	Alzheimer’s disease	Aducanumab	0.9932	0.7648	8.1E-6	10/02/01	0.0059
Vipadenant	Parkinson’s disease	Donanemab	0.9916	0.7175	0.0002	NA	NA
Lemon oil	Anxiety	Rivastigmine	0.9885	0.7091	0.0002	NA	NA
**Latrepirdine**	Alzheimer’s disease	Rivastigmine	0.9929	0.7049	0.0003	05/09/01	0.0051
**Mecamylamine**	Hypertension	Galantamine	0.9944	0.7043	0.0003	20/03/15	0.0015
**Acamprosate**	Alcohol dependence	Brexpiprazole	0.9967	0.7042	0.0003	13/02/01	0.0007
Madopar	Parkinson’s disease	Memantine	0.9917	0.7029	0.0003	NA	NA
**Pimavanserin**	Hallucinations	Brexpiprazole	0.9951	0.7024	0.0003	13/11/01	0.0008
**ST-101**	Alzheimer’s disease	Rivastigmine	0.9901	0.7006	0.0003	09/02/01	NA
Reboxetine	Depression	Brexpiprazole	0.9951	0.6985	0.0004	NA	NA
Valerian	Anxiety	Galantamine	0.9911	0.6978	0.0004	NA	NA
Cariprazine	Schizophrenia	Brexpiprazole	0.9976	0.6895	0.0006	NA	NA
**Baclofen**	Spasticity	Memantine	0.9913	0.6889	0.0006	13/02/01	0.0005
5-HTP	Depression	Galantamine	0.9916	0.6882	0.0006	NA	NA
**Psilocybin**	Anxiety	Brexpiprazole	0.9963	0.6795	0.0008	21/03/24	0.0027
Cytisine	Smoking cessation	Galantamine	0.9954	0.6789	0.0009	NA	NA
Lurasidone	Schizophrenia	Brexpiprazole	0.9961	0.6788	0.0009	NA	NA
**Lithium**	Depression	Donepezil	0.9938	0.6743	0.0010	04/07/01	0.0010
Liatermin	Parkinson’s disease	Aducanumab	0.9875	0.6737	0.0010	NA	NA
Blonanserin	Schizophrenia	Brexpiprazole	0.9962	0.6660	0.0014	NA	NA
Tozadenant	Parkinson’s disease	Memantine	0.9871	0.6557	0.0020	NA	NA
Triumeq	HIV-1 infection	Aducanumab	0.9888	0.6543	0.0021	NA	NA
**Rasagiline**	Parkinson’s disease	Lecanemab	0.9896	0.6465	0.0027	04/08/01	NA
ITI-214	Parkinson’s disease	Galantamine	0.9928	0.6368	0.0037	NA	NA
Adiphenine	Muscle spasms	Brexpiprazole	0.9915	0.6094	0.0082	NA	NA

*: not necessary an approved indication.

**: date format: YY/MM/DD. Correlation: the Pearson correlation coefficient determined by comparing the drug and its closest ADRD drug, as per the R-score profile.

**Table 3.** Example outcomes of different hypothesis generation tasks. The reference numbers in this table correspond to the indices of the input sentences in the prompts.

Task	Hypothesis
Generating a hypothesis based on supporting sentences (”LitKG-GPT4”)	Based on the evidence presented, it can be hypothesized that phenserine may treat Alzheimer’s disease (AD) by reducing the production of amyloid precursor protein and beta-amyloid, key components in the formation of amyloid plaques, a hallmark of AD [23, 31, 79]. Phenserine has been shown to significantly increase doublecortin immunoreactivity within the subventricular zone of AD mice, suggesting a potential role in neurogenesis [55]. Additionally, phenserine has been found to be a reversible acetylcholinesterase inhibitor, which could potentially alleviate the decreased acetylcholinesterase activity observed in AD [51]. Furthermore, phenserine has demonstrated the ability to reduce amyloid-beta concentrations in cell culture, mice, and humans [21]. In a clinical trial, phenserine reduced cerebrospinal fluid levels of amyloid-beta and the formation of amyloid plaques [85]. Therefore, the therapeutic effect of phenserine in AD may be attributed to its multifaceted actions on amyloid-beta production, acetylcholinesterase inhibition, and potential promotion of neurogenesis [21,23, 55,85].
Generating a hypothesis based on supporting sentences and unrelated sentences (”LitKG’-GPT4”)	Phenserine, a reversible acetylcholinesterase (AChE) inhibitor, has been shown to possess neuroprotective properties and the ability to reduce amyloid precursor protein (APP) levels, which are implicated in the pathogenesis of Alzheimer’s disease (AD) [88]. The reduction of APP synthesis and A*β*42 concentrations by phenserine has been demonstrated in cell culture, mice, and humans [21]. This is significant as the aggregation and misfolding of amyloid-beta (A*β*) is a potential mechanism in AD, with A*β* oligomers being neurotoxic and highly correlated with severe AD [15]. Furthermore, the overexpression of APP and the subsequent production of A*β* have been linked to early-onset AD [4, 22, 74, 77]. Phenserine’s ability to inhibit AChE activity is also relevant, as decreased AChE activity has been observed in AD [51]. Moreover, phenserine has been shown to increase doublecortin reactivity in the subventricular zone of AD mice, suggesting a potential role in neurogenesis [55]. Therefore, it can be hypothesized that phenserine may treat AD by reducing APP levels and A*β* production, inhibiting AChE activity, and promoting neurogenesis.
Generating a hypothesis based on relation types and unrelated relations (”KG-GPT4”)	Phenserine, a drug known to inhibit amyloid precursor protein biosynthetic process and Amyloid beta-Peptides, may have potential therapeutic effects in the treatment of Alzheimer’s disease [21, 23]. Amyloid beta-Peptides are known to cause Alzheimer’s disease and are associated with various forms of the disease [12, 22, 25, 28, 39, 42, 53, 54, 68, 74, 77, 83, 87]. Furthermore, the biosynthetic process of amyloid precursor protein coexists with Alzheimer’s disease [26]. Phenserine also inhibits acetylcholinesterase activity [86], which is found to coexist with Alzheimer’s disease [4, 5, 51, 49]. Additionally, phenserine stimulates immunoreactivity [55], which is also associated with Alzheimer’s disease [35, 37, 56, 89]. Therefore, the inhibition of amyloid precursor protein biosynthetic process and Amyloid beta-Peptides, along with the inhibition of acetylcholinesterase activity and stimulation of immunoreactivity by phenserine, could potentially contribute to its therapeutic effects in Alzheimer’s disease.
Generating a hypothesis without further information (”GPT4”)	Phenserine, a reversible acetylcholinesterase inhibitor, may have potential therapeutic effects in the treatment of Alzheimer’s disease through several mechanisms. The primary hypothesis is that Phenserine can increase the levels of acetylcholine, a neurotransmitter that is significantly reduced in Alzheimer’s disease, by inhibiting its breakdown. This could potentially improve cognitive function and memory in patients. Additionally, Phenserine may also exert neuroprotective effects by reducing the production of amyloid-beta peptides, which are known to accumulate in the brains of Alzheimer’s patients and contribute to neuronal damage and cognitive decline. Furthermore, Phenserine might also modulate other pathological processes involved in Alzheimer’s disease, such as oxidative stress and inflammation, thereby slowing disease progression. However, these hypotheses need to be confirmed through rigorous clinical trials.
Generating a hypothesis by Consensus without further information (”Consensus”)	Phenserine, an acetylcholinesterase (AChE) inhibitor, holds promise for treating Alzheimer’s disease (AD) through both cholinergic and non-cholinergic mechanisms. As an AChE inhibitor, phenserine increases acetylcholine levels, enhancing cognitive function by improving neurotransmission. Additionally, phenserine has been shown to reduce the production of amyloid-beta (A*β*) by modulating the translation of amyloid precursor protein (APP) mRNA, thereby lowering A*β* levels, which are implicated in the pathogenesis of AD (Winblad et al., 2011). In clinical studies, phenserine has demonstrated cognitive improvement and a reduction in brain amyloid load, suggesting its potential to modify disease progression rather than merely alleviating symptoms (Kadir et al., 2008). These dual actions position phenserine as a multifaceted candidate for AD therapy, targeting both symptomatic relief and underlying disease processes.

## Data Availability

The datasets used and/or analysed during the current study available from the corresponding author on reasonable request.
